# Hepatitis B virus (HBV) viral load, liver and renal function in adults treated with tenofovir disoproxil fumarate (TDF) vs. untreated: a retrospective longitudinal UK cohort study

**DOI:** 10.1186/s12879-021-06226-0

**Published:** 2021-06-26

**Authors:** Tingyan Wang, David A. Smith, Cori Campbell, Jolynne Mokaya, Oliver Freeman, Hizni Salih, Anna L. McNaughton, Sarah Cripps, Kinga A. Várnai, Theresa Noble, Kerrie Woods, Jane Collier, Katie Jeffery, Jim Davies, Eleanor Barnes, Philippa C. Matthews

**Affiliations:** 1 Nuffield Department of Medicine, University of Oxford, Oxford, UK; 2 National Institute for Health Research (NIHR), Oxford Biomedical Research Centre, Oxford, UK; 3 NIHR Health Informatics Collaborative, Oxford University Hospitals NHS Foundation Trust, Oxford, UK; 4 Nuffield Department of Population Health, University of Oxford, Oxford, UK; 5 Pharmacy Department, John Radcliffe Hospital, Oxford University Hospitals NHS Foundation Trust, Oxford, UK; 6 Department of Hepatology, John Radcliffe Hospital, Oxford University Hospitals NHS Foundation Trust, Oxford, UK; 7 Department of Infectious Diseases and Microbiology, John Radcliffe Hospital, Oxford University Hospitals NHS Foundation Trust, Oxford, UK; 8 Department of Computer Science, University of Oxford, Oxford, UK

**Keywords:** Liver fibrosis, eGFR, Tenofovir Disoproxil fumarate (TDF) therapy, Chronic hepatitis B, Renal impairment

## Abstract

**Background:**

Current clinical guidelines recommend treating chronic hepatitis B virus (HBV) infection in a minority of cases, but there are relatively scarce data on evolution or progression of liver inflammation and fibrosis in cases of chronic HBV (CHB) that do not meet treatment criteria. We aimed to assess the impact of TDF on liver disease, and the risk of renal impairment in treated CHB patients in comparison to untreated patients.

**Methods:**

We studied a longitudinal ethnically diverse CHB cohort in the UK attending out-patient clinics between 2005 and 2018. We examined TDF treatment (vs. untreated) as the main exposure, with HBV DNA viral load (VL), ALT, elastography scores and eGFR as the main outcomes, using paired tests and mixed effects model for longitudinal measurements. Additionally, decline of eGFR during follow-up was quantified within individuals by thresholds based on clinical guidelines. Baseline was defined as treatment initiation for TDF group and the beginning of clinical follow-up for untreated group respectively.

**Results:**

We included 206 adults (60 on TDF, 146 untreated), with a median ± IQR follow-up duration of 3.3 ± 2.8 years. The TDF group was significantly older (median age 39 vs. 35 years, *p* = 0.004) and more likely to be male (63% vs. 47%, *p* = 0.04) compared to the untreated group. Baseline difference between TDF and untreated groups reflected treatment eligibility criteria. As expected, VL and ALT declined significantly over time in TDF-treated patients. Elastography scores normalised during treatment in the TDF group reflecting regression of inflammation and/or fibrosis. However, 6/81 (7.4%) of untreated patients had a progression of fibrosis stage from F0-F1 to F2 or F3. There was no evidence of difference in rates or incidence of renal impairment during follow-up in the TDF vs. untreated group.

**Conclusions:**

Risk of liver inflammation and fibrosis may be raised in untreated patients compared to those receiving TDF, and TDF may benefit a larger percentage of the CHB population.

**Supplementary Information:**

The online version contains supplementary material available at 10.1186/s12879-021-06226-0.

## Background

The World Health Organisation (WHO) estimates that only 10.5% of the 257 million individuals living with chronic HBV infection are aware of their infection, and that among these, < 20% are receiving antiviral treatment [1]. Nearly one million annual deaths are attributable to HBV, resulting largely from progression to cirrhosis and hepatocellular carcinoma (HCC). Curing chronic HBV infection (CHB) is challenging due to the persistence of covalently closed circular DNA (cccDNA) in infected hepatocytes [2]. However, treatment with nucleot(s)ide analogues (NAs) can suppress viral replication, thus reducing the risk of inflammatory liver disease, cirrhosis and HCC [2, 3]. HBV surface antigen (HBsAg) loss, sometimes termed ‘functional cure’, is regarded as the optimal endpoint of current treatment in clinical guidelines, but is infrequently achieved, as evidenced by the annual rate of HBsAg loss ranging from 0.12 to 2.7% [4–6]. Therefore, NA therapy is typically long-term, and can be life-long [2].

Existing CHB guidelines [2, 3, 7] recommend stratifying patients for therapy based on demographic, clinical and laboratory parameters, such that only a minority (e.g., ranging from 2% ~ 31% in different settings [8–10]) are treatment-eligible. Tenofovir disoproxil fumarate (TDF) is an affordable, well-tolerated NA that has a high genetic barrier to resistance, and is thus the most commonly used first-line option for CHB patients. Long-term efficacy of TDF has been confirmed by extended follow-up, demonstrating viral suppression [11], improvement in liver histology in patients with baseline cirrhosis [12, 13], and reduced cumulative probability of HCC, hepatic decompensation, death and liver transplantation, compared to non-treatment [14]. Although previous studies provide evidence for the beneficial association of TDF treatment with HCC or cirrhosis risk, the influence of TDF on the evolution or progression of liver inflammation and fibrosis in non-cirrhotic HBV patients is less well understood [15].

Benefits of TDF therapy must be balanced with concerns about potential risks, of which the most widely recognised is renal toxicity [2]. However, the long-term renal safety of TDF in CHB patients remains controversial [15–20]. Most evidence for TDF-mediated renal injury comes from human immunodeficiency virus (HIV) cohorts, in which associations may be confounded by other drugs, comorbidities, or HIV-associated nephropathy [21, 22]. HBV guidelines produced by the European Association for the Study of the Liver (EASL) recommend that all TDF-treated patients undergo renal monitoring [2]. One study carried out among CHB patients with high HBV DNA viral load (> 6 log_10_ IU/ml) reported that TDF treatment was associated with a higher incidence of acute kidney injury (AKI) compared to ETV, but this difference was only borderline significant after three years follow-up [16]. Similar findings have been documented in other cohorts [17]. In patients with baseline renal impairment and diabetes mellitus, TDF may increase the risk of renal function decline [23, 24]. However, this observation is not consistent, as other studies have failed to identify significant difference in the risk of renal events between TDF- and ETV-treated patients [15, 18, 19], and have not reported deteriorations in renal function on TDF therapy [20] particularly if there is no renal impairment at baseline [24]. Heterogenous findings regarding the risk of renal toxicity for TDF-treated patients may be due to differences in the characteristics of patient cohorts in terms of age, estimated glomerular filtration rate (eGFR) at baseline, and prevalence of other chronic health conditions such as hypertension and diabetes [25, 26].

Overall, most previous studies have focused on longitudinal comparison of CHB patients treated with TDF vs. non-TDF agents [11–20, 24, 27], but studies comparing changes of liver and/or renal function over time in TDF treated vs. untreated populations are limited [23, 28, 29]. Furthermore, previous studies have typically only compared average efficacy or renal safety of TDF at a population level at each time point, rather than examining longitudinal changes in liver or renal function at the individual patient level. The latter can avoid ecological fallacy and provide more meaningful insights to help identify untreated CHB cases who might benefit from treatment.

To examine the influence of TDF on risk of inflammatory liver disease, and to assess the renal safety of TDF in CHB patients, we analysed longitudinal changes in liver enzymes, elastography scores, and renal function both at a population- and individual-level, comparing TDF-treated patients to treatment-naïve patients. Specifically, we investigated: a) the effects of TDF treatment on maintenance of viral suppression, and reversal of liver inflammation and fibrosis, and b) the risks of renal toxicity associated with TDF treatment in CHB patients with mild/moderate liver disease at baseline.

## Methods

### Study cohort

We conducted a longitudinal retrospective study on an adult CHB cohort in the United Kingdom between 06/2005 and 10/2018. Data were collected from Oxford University Hospitals (OUH) National Health Service (NHS) Foundation Trust, a large teaching hospital trust in the South East of the UK, using a clinical informatics pipeline supported by the National Institute for Health Research Health Informatics Collaborative (NIHR HIC) [6, 30].

We examined TDF treatment (vs. untreated) as the main exposure, with HBV DNA viral load (VL), ALT, elastography score and eGFR as the main outcomes. Baseline is defined as the start date of treatment for TDF group and the beginning of clinical follow-up for the untreated group. We planned to censor patients if they were lost to follow-up, died, or met exclusion criteria during follow-up, whichever occurred first. In practice, the only event in this category was loss to follow-up. The inclusion criteria were: (1) patients with CHB (defined as two positive HBsAg tests and/or detectable HBV DNA tests at least 6 months apart); and (2) patients on TDF monotherapy or treatment naïve patients (i.e., individuals who were not on treatment with NAs or interferon).

The exclusion criteria were: (1) patients with hepatitis C virus (HCV), hepatitis delta virus (HDV) or HIV coinfection; (2) patients with decompensated cirrhosis at baseline; (3) patients who had HCC at baseline; (4) patients who were younger than 18 years at baseline; (5) patients who had been followed-up for less than 1 year from baseline; or (6) patients with < 2 measurements of eGFR.

### Laboratory markers

Quantitative HBV DNA testing was undertaken at OUH NHS Foundation Trust clinical diagnostic microbiology laboratory using the Cobas TaqMan assay (Roche Diagnostics, Branchburg, NJ), with a lower limit of detection of 9 IU/ml. Serum HBV e-antigen (HBeAg) was measured using Centaur (09/2004–12/2014) or Abbott Architect i2000SR (Abbott Laboratories, Chicago, IL) (12/2014–10/2018). Quantitative HBsAg was tested using Centaur (09/2004–12/2014) or Abbott Architect i2000SR (Abbott Laboratories, Chicago, IL) (12/2014–10/2018) with a lower limit of detection of 0.05 IU/ml. Alanine transaminase (ALT) was tested using Siemens ADVIA 2400 (02/2013–01/2015) or Abbott Architect c16000 or c8000 (Abbott Laboratories, Chicago, IL) (01/2015–10/2018) with the normal reference range from 10 to 45 IU/L. Our clinical diagnostic laboratory does not set different ALT reference ranges for males and females. Creatinine was measured in micromoles per litre (μmol/l) in this study with a normal reference range of 64 to 104 μmol/l.

We defined: (a) virologic response as detectable serum HBV DNA VL at baseline which is suppressed to < 20 IU/ml during the follow-up, and after which there is no increase of ≥ 1 log_10_ IU/ml from the nadir level achieved [2]; (b) HBeAg loss as a change from being HBeAg positive to HBeAg negative; (c) HBeAg seroconversion as the new detection of antibody to HBeAg (anti-HBe); (d) HBsAg loss as negative HBsAg or undetectable HBsAg by quantitative test following a previously positive result; (e) biochemical responses as the normalisation of ALT levels (i.e., decline of baseline ALT levels to ≤ 45 IU/L and maintaining this level during the follow-up period).

### Liver fibrosis assessment

We evaluated transient elastography (TE) (or FibroScan), a non-invasive test measuring liver stiffness in kiloPascals (kPa), as a marker of liver inflammation and/or fibrosis [31]. No universally-agreed thresholds are available to map TE values to histological METAVIR stages [32], but TE is well-validated in both HCV and HBV and cut-off values have been suggested in these populations [33, 34]. We used the following thresholds for fibrosis stages: F0 with TE score of < 7.0 kPa, F1 with TE score of ≥ 7.0 kPa to < 8.0 kPa, F2 with TE score of ≥ 8.0 kPa to < 10.0 kPa, F3 with TE score of ≥ 10.0 kPa to < 14.0 kPa, and F4 with TE score of ≥ 14 kPa [34].

### Renal function assessment

eGFR is used to measure renal function, which was directly reported by our clinical laboratory with a unit of ml/min/1.73m^2^. During the study period, the Modification of Diet in Renal Disease (MDRD) Study equation was used by our laboratory for eGFR calculation. eGFR is typically classified into five categories, G1-G5, used to monitor the development or progression of chronic kidney disease (CKD) [35], as follows:

- Normal kidney function defined as eGFR ≥ 90 ml/min/1.73m^2^ (G1);
- Mildly decreased kidney function if eGFR 60–89 ml/min/1.73m^2^ (G2);
- Mild-moderate loss of kidney function if eGFR 45–59 ml/min/1.73m^2^ (G3a);
- Moderate-severe loss of kidney function if eGFR 30–44 ml/min/1.73m^2^ (G3b);
- Severe loss of kidney function if eGFR 15–29 ml/min/1.73m^2^ (G4);
- Kidney failure if eGFR < 15 ml/min/1.73m^2^ (G5).

Adverse events related to AKI are defined as an elevation in serum creatinine of ≥ 0.3 mg/dl (26.5 μmol/l in this study) or ≥ to 1.5 times from a known or presumed baseline based on AKI clinical guidelines [36–38].

### Ethnicity data

Ethnicity was self-reported according to NHS standard ethnic category code list [39], and we used the following top-level categories: “Asian”, “Black”, “Mixed”, “White", or “Other”; ethnic groups.

### Data extraction

As data used in this study were collected via a pipeline supported by NIHR HIC and stored in a central database [30], we used structured query language (SQL) techniques for data retrieval and extraction. We focused on longitudinal changes in biomarkers, and therefore for each patient we collected data at baseline and at multiple subsequent follow-up time points at 3, 6, 12, 18, 24, 30, 36, 42 and 48 months. As this is routinely collected clinical data, the time intervals of follow-up were not regular with missing data at certain time points. To fully use the irregular data for longitudinal change pattern analysis, we used an imputation scheme, i.e., if the data at a certain time point *t* was missing, we used the data closest to that time point within *t* ± 1.5 or 3 months (Fig. S1).

### Statistical analysis

We conducted all statistical analyses in R (version: 3.6.1). Significance tests were two-sided and *p* values < 0.05 were deemed statistically significant. We used Fisher’s exact test for categorical variables, while for continuous variables we performed Wilcoxon rank sum test. Log transformation was used for HBV DNA VL, and ALT values were transformed into fold of the upper limit of normal (ULN). To examine the efficacy of TDF on VL suppression (or ALT normalisation), we compared the VL (or ALT level) at different time points after treatment to VL (or ALT level) at baseline by using paired t-test or paired Wilcoxon test as appropriate, depending on the size of samples or the result of normality test by Shapiro-Wilk’s test. We applied the Kaplan-Meier (K-M) method to estimate the rates of virologic response over time. We used log rank tests to compare the difference in the cumulative rates of HBV DNA VL suppression or ALT normalisation between subgroups. As censoring could influence interpretation of K-M curves, we marked censored subjects on the curve by points. If the event of interest (i.e., HBV DNA VL suppression, ALT normalisation) did not occur before the end of follow-up, this patient was censored (the total time to an event for this subject could not be accurately determined) [40].

To further evaluate and compare the changes of HBV DNA VL, ALT, TE, and eGFR over time between TDF and untreated groups, we used linear mixed effects models for repeated measures [41, 42] using the lme4 package (version: 1.1-23) [43]. Linearity assumption was checked by residual plot. If the linearity assumption was not met, we applied a nonlinear transformation [42]. In the crude (unadjusted) models, we considered follow-up time, group, and group-by-time interaction as fixed effects and incorporated random intercepts for individuals. We used the likelihood ratio test to evaluate whether it is necessary to add a random slope for time to a model [41]. We then adjusted for baseline age, gender, and ethnicity in these models. We also performed sensitivity analysis to include further adjustment for other imbalanced baseline characteristics if these data were available.

We stratified TE scores into different categories for matching METAVIR stages at each time point. To examine the progression of liver fibrosis, we analysed the changes of TE score categories for individuals from baseline to the end of follow-up. To quantify the longitudinal variability of eGFR within individuals, we used the standard deviation (SD) of eGFR during the follow-up period for each patient, which measures the amplitudes of eGFR values from mean eGFR level within an individual. Furthermore, to examine changes in renal function, we stratified the total decline of eGFR levels during the follow-up within individuals into three categories relative to baseline or the first available observation, i.e., decline greater than 10 ml/min/1.73m^2^, decline less then 10 ml/min/1.73m^2^ and no decline, based on the definition published by NICE guidelines [35]. To examine the progression of CKD, we analysed the drop of eGFR categories (G1, G2, G3a, G3b, G4, G5 as defined). To assess probable AKI events, we stratified the maximum change amplitude of serum creatinine levels during the follow-up into three different elevation levels based on AKI clinical guidelines [36–38], i.e., (a) elevation ≥ 26.5 μmol/l or ≥ 1.5 times, (b) elevation < 26.5 μmol/l and < 1.5 times, and (c) no elevation. The maximum change amplitude here is defined as the maximal value of changes in serum creatinine levels between every two consecutive tests within a patient.

## Results

The detailed information for selecting patients based on each criterion is illustrated in a flow diagram (Fig. S2). We included 206 patients (60 patients treated with TDF monotherapy, and 146 patients without treatment). All patients had at least two measurements of ALT, eGFR, and serum creatinine. 202/206 patients had at least two measurements of HBV DNA VL.

### Patients treated with TDF are older with more prevalent liver fibrosis compared to the untreated group at baseline

We compared baseline characteristics for individuals treated with TDF vs. non-treated (Table 1). There was no significant difference in the length of follow-up between the two groups (*p* = 0.2). Treated patients were significantly older than those untreated (median 39 vs. 35 years, respectively, *p* = 0.004), with more males (63% vs. 47%, *p* = 0.04). As expected, based on stratification for treatment using national guidelines [7], patients treated with TDF were more likely to have raised ALT (*p* < 0.001), higher HBV DNA VL (p < 0.001), and higher TE scores (*p* = 0.002) at baseline. However, there was no difference in baseline renal function, based either on eGFR, or creatinine.

**Table 1:** Baseline characteristics of adults with chronic hepatitis B virus infection (Tenofovir disoproxil fumarate vs. untreated)

	TDF group	Untreated group	***p***-value
	(***n*** = 60)	(***n*** = 146)	
Follow-up duration (years)	3.8 [2.6, 4.6]	3.0 [1.9, 4.9]	0.176
Age (years)	39 [33, 48]	35 [30, 40]	**0.004**
Age > 60 (years) (%)	4 (6.7)	5 (3.4)	0.510
Gender (%)			
Female	20 (33.3)	71 (48.6)	0.064
Male	38 (63.3)	68 (46.6)	**0.042**
Unreported	2 (3.3)	7 (4.8)	1
Ethnicity (%) ^§^			
Asian	33 (55.0)	33 (22.6)	**< 0.001**
Black	11 (18.3)	31 (21.2)	0.780
White	9 (15.0)	28 (19.2)	0.610
Other	4 (6.7)	10 (6.8)	1
Unreported	3 (5.0)	44 (30.1)	**< 0.001**
HBeAg positive (%)	5 (8.3)	4 (2.7)	0.126
Diabetes (%) ^†^			
Yes	2 (3.3)	6 (4.1)	1
No	25 (41.7)	72 (49.3)	0.398
Unknown	33 (55.0)	68 (46.6)	0.344
ALT (x ULN) (IU/L)	1.1 [0.7, 1.8]	0.6 [0.4, 0.8]	**< 0.001**
HBV DNA (log_10_ IU/ml)	4.8 [4.1, 6.1]	2.7 [1.9, 3.5]	**< 0.001**
qHBsAg (log_10_ IU/ml)	3.0 [3.0, 3.6]	3.0 [3.0, 3.6]	0.425
Liver stiffness (kPa) ^‡^	7.5 [5.0, 10.0]	5.0 [4.0, 6.0]	**0.002**
< 8 kPa	11 (18.3)	69 (47.3)	**< 0.001**
≥ 8 kPa and < 14 kPa	9 (15.0)	6 (4.1)	**0.015**
≥ 14 kPa	2 (3.3)	0 (0.0)	**< 0.001**
Unavailable	38 (63.3)	71 (48.6)	0.077
eGFR (ml/min/1.73m^2^) ^#^	90 [89, > 90]	90 [87, > 90]	0.613
≥ 90 ml/min/1.73m^2^	29 (48.3)	72 (49.3)	1
60 ~ 89 ml/min/1.73m^2^	11 (18.3)	30 (20.5)	0.865
< 60 ml/min/1.73m^2^	0 (0)	1 (0.97)	1
Unavailable	20 (33.3)	43 (29.5)	0.702
Serum creatinine (μmol/l)	72 [57, 80]	73 [57, 83]	0.766
Serum urea (mmol/l)	5 [4, 6]	4 [4, 6]	0.199
Albumin (g/L)	42 [38, 44]	41 [38, 45]	0.861
ALP (IU/L)	114 [63, 163]	96 [63, 150]	0.266
Bilirubin (total) (umol/L)	10 [8, 15]	9 [7, 12]	0.104
Platelet count (× 10^9^/L)	205 [162.5, 240.0]	219 [185.5, 252.2]	0.078

Data are the median [interquartile range] or number (%) unless otherwise indicated. For categorical variables, Fisher's exact test was performed for comparison on cells with small counts (< 5), otherwise Chi-square test was used. For continuous variables, Wilcoxon test was used for comparison due to non-normality. *p* values < 0.05 were deemed statistically significant, marked in bold. ^§^ Ethnicity was originally self-reported by patients according to NHS standard ethnic categories. ^†^ Diabetes was diagnosed using glycated haemoglobin (HbA1c), an HbA1c of 6.5% or 47.5 mmol/mol is a typical threshold for diagnosing diabetes. ^‡^ Liver stiffness was measured by transient elastography score in kiloPascals (kPa). ^#^ For eGFR, if a level was greater than 90 ml/min/1.73m^2^, it was not quantified by the hospital laboratory system. *ALT* Alanine aminotransferase; *eGFR* estimated Glomerular Filtration Rate; *ALP* Alkaline phosphatase; *TDF* Tenofovir disoproxil fumarate; *qHBsAg* quantitative HBsAg level*; ULN* upper limit of normal

Interestingly, a higher TDF treatment rate was observed in Asian patients compared to patients from other ethnic groups (55.0% vs. 22.6%, p < 0.001). However, ethnicity was unreported in 47 (22.8%) patients (Table 1). With analysis stratified by ethnicity (Table S1), we found a significantly higher proportion of patients aged > 60 years, significantly higher ALT levels (*p* = 0.01), and higher HBV DNA VL (*p* = 0.02) at baseline in Asian patients, compared to patients of other ethnicities. These findings may also be in keeping with a higher HBeAg-positive prevalence amongst Asian patients but HBeAg status was missing in 14.3–40.9% of cases (Table S1), limiting our ability to make meaningful comparisons.

### The majority of patients on TDF treatment suppressed VL within 12 months, while untreated individuals maintained a virological set point during follow-up

**Fig. 1:**
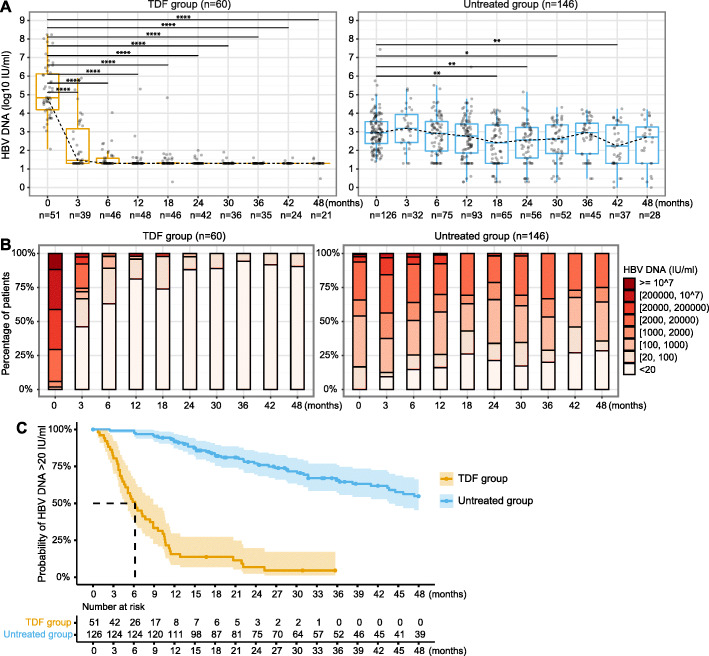
Longitudinal analysis of HBV DNA VL in chronic HBV patients with TDF treatment vs. without treatment. (**A)** Distribution of HBV DNA VL at each time point; (**B**) Stratification of HBV DNA VL at each time point; (**C**) Kaplan-Meier analysis to estimate the probability of patients with HBV DNA VL > 20 IU/ml in each group over time. In Kaplan-Meier analysis, real dates were used for calculating the time to HBV DNA VL suppressed < 20 IU/ml, rather than the imputed time points and censored subjects were marked on the curve by dots. * p-value < 0.05, ** p-value < 0.01, *** p-value < 0.001, **** p-value < 0.0001. HBV, hepatitis B virus; TDF, Tenofovir disoproxil fumarate; VL, viral load

In the TDF group, a significant decline was observed in a pairwise comparison of VL at baseline compared to all later time points (all *p* < 0.0001, Fig. 1A, left hand panel). In the untreated group, a significant difference was only found in the pairwise comparison of VL at selected later time points compared to baseline VL and there was no significant difference between VL at baseline and 12 months, or between baseline and the final timepoint of 48 months (Fig. 1A, right hand panel). The crude mixed effects model further revealed that changes in VL over time were significantly different between the TDF-treated and untreated groups (*p* < 0.001, Table 2) with a faster decline over time in the TDF-treated group, as expected. Similar longitudinal trends of VL were obtained from the model adjusted for demographics (Table 2) and sensitivity analysis with further adjustment for baseline ALT and TE scores (Table S2).

**Table 2:** Changes of HBV DNA VL (log_10_ IU/ml) over time assessed by linear mixed effects models for patients treated with TDF vs. untreated

	Crude model		Adjusted for baseline age, gender, ethnicity	
	Coefficient β (95% CI)	***p***-value	Coefficient β (95% CI)	***p***-value
(Intercept)	2.783 (2.627, 2.939)	**< 0.001**	﻿3.476 (﻿3.020, 3.931)	**< 0.001**
Group	0.162 (− 0.114, 0.437)	0.250	0.123 (− 0.163, 0.409)	0.399
Time	−0.013 (− 0.019, − 0.007)	**< 0.001**	−0.013 (− 0.019, − 0.007)	**< 0.001**
Group x Time ^**a**^	−0.041 (− 0.050, − 0.032)	**< 0.001**	− 0.041 (− 0.050, − 0.032)	**< 0.001**

^**a**^ x indicates the interaction between group and follow-up time. Group = {0,1}, where 0 indicates untreated, 1 indicates TDF. A log transformation was applied to the dependent variable (HBV DNA) due to nonlinearity. *CI* confidence interval; *TDF* Tenofovir disoproxil fumarate; *VL* viral load

In the TDF group, 81.3% of patients suppressed VL to < 20 IU/ml at 1 year, 88.1% at 2 years, and 94.1% at 3 years, while all had VL < 2 log_10_ IU/ml at the end of follow-up (Fig. 1B). In contrast, only ~ 25% of untreated patients had VL < 20 IU/ml at one to 3 years, with medians (±IQR) of 2.8 ± 1.5 log_10_ IU/ml, 2.6 ± 1.6 log_10_ IU/ml, and 3.0 ± 1.6 log_10_ IU/ml at 1 year, 2 years, and 3 years, respectively. At the end of follow-up only 36% of the untreated group had VL < 2 log_10_ IU/ml (Fig. 1B).

Kaplan-Meier analysis further estimated that the median time to virologic suppression was 6 months for the TDF group (Fig. 1C). 51/60 (85%) patients in the TDF group vs. 126/146 (86.3%) patients in the untreated group had baseline VL data (*p* = 0.8), and all these patients had VL > 20 IU/ml at baseline. Among these patients, 94.1% (48/51) in the TDF group vs. 34.9% (44/126) in the untreated group had suppressed VL to < 20 IU/ml during follow-up (*p* < 0.001). The remaining 3 (3/51, 5.9%) TDF-treated patients with VL > 20 IU/ml were censored at 17, 31, and 36 months. The remaining 81 (81/126, 65.1%) untreated patients with detectable VL were censored at a mean of 3.7 years, and none of them started treatment during follow-up.

An increase of VL by > 1 log_10_ IU/ml during follow-up was observed in 2/60 (3.3%) in the TDF group vs. 25/146 (17.1%) in the untreated group (*p* = 0.006) (Fig. S3 and Fig. S4). In the untreated group, there is evidence of a set-point HBV DNA VL, where 17.3% of variations in VL were accounted for by within-patient variation, compared to 82.7% between patients, as previously described [44].

### Patients treated with TDF have higher baseline ALT levels that normalise with treatment

**Fig. 2:**
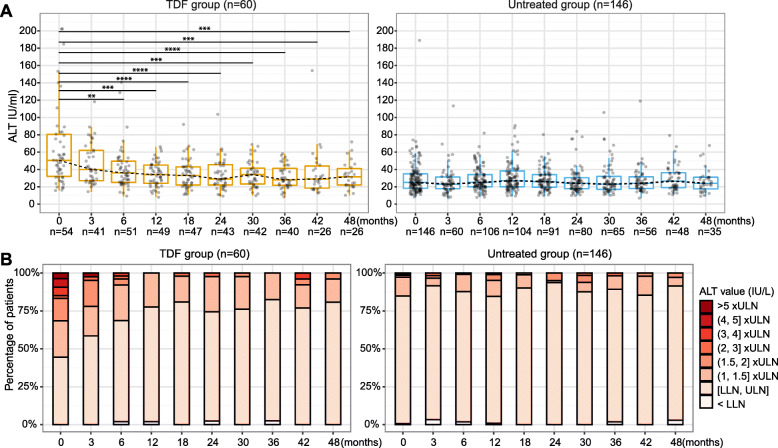
Longitudinal analysis of ALT of chronic HBV patients with TDF treatment vs. without treatment. (**A**) Distribution of ALT levels at each time point; (**B**) Stratifications of ALT levels at each time point. * p-value < 0.05, ** p-value < 0.01, *** p-value < 0.001, **** p-value < 0.0001. ALT, Alanine aminotransferase; TDF, Tenofovir disoproxil fumarate

In the TDF group, ALT levels progressively normalised after treatment initiation, and significant differences were observed at six months and at later timepoints with a pairwise comparison to baseline ALT levels (all *p* < 0.01, Fig. 2A). In the untreated group, no differences were found for ALT at any later timepoints compared to baseline levels (Fig. 2A). Furthermore, mixed effects models showed that changes of ALT over time were significantly different for TDF-treated and untreated groups (*p* < 0.001, Table 3) with a faster decrease over time in TDF-treated group; consistent findings were obtained from sensitivity analysis (Table S3). In the treated group, > 70% of patients had ALT levels within the normal range at 12 months and later timepoints, while ~ 85% in untreated group had normal ALT level at each time point (Fig. 2B).

**Table 3:** Changes of ALT (IU/L) over time assessed by linear mixed effects models for patients treated with TDF vs. untreated

	Crude model		Adjusted for baseline age, gender, ethnicity	
	Coefficient β (95% CI)	***p***-value	Coefficient β (95% CI)	***p***-value
(Intercept)	30.182 (26.944, 33.421)	**< 0.001**	44.491 (35.438, 53.545)	**< 0.001**
Group	23.566 (17.691, 29.440)	**< 0.001**	21.868 (16.058, 27.677)	**< 0.001**
Time	−0.055 (− 0.169, 0.059)	0.346	− 0.055 (− 0.168, 0.059)	0.347
Group x Time ^**a**^	− 0.562 (− 0.752, − 0.372)	**< 0.001**	−0.579 (− 0.768, − 0.390)	**< 0.001**

^**a**^ x indicates the interaction between group and follow-up time. Group = {0,1}, where 0 indicates untreated, 1 indicates TDF. *ALT* Alanine aminotransferase; *CI*,confidence interval; *TDF* Tenofovir disoproxil fumarate

Although the proportion of patients who had abnormal ALT levels at baseline was significantly higher in the TDF group (30/60, 50.0%) compared to the untreated group (22/146, 15.1%) (*p* < 0.0001), ALT normalisation was observed in both these groups during follow-up, i.e., 93.3% (28/30) in treated patients and 86.4% (19/22) in untreated patients (*p* = 1). The remaining two (2/30, 6.7%) TDF-treated patients with abnormal ALT were censored at 35 and 56 months as time to ALT normalisation could not be evaluated. The remaining three (3/22, 13.6%) untreated patients with abnormal ALT were censored at 12, 56, and 66 months, and none of them started treatment during follow-up.

### TE score regressed in patients treated with TDF and progressed in untreated patients

**Fig. 3:**
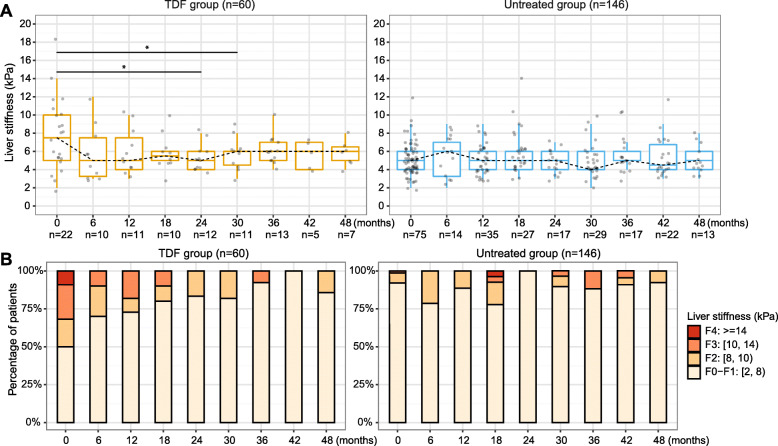
Longitudinal analysis of liver stiffness (transient elastography scores) of chronic HBV patients with TDF treatment vs. without treatment. **A** Distribution of transient elastography scores at each time point; (**B**) Stratifications of transient elastography scores at each time point. * p-value < 0.05, ** p-value < 0.01, *** p-value < 0.001, **** p-value < 0.0001. TDF, Tenofovir disoproxil fumarate

Baseline TE scores were available for 22/60 (36.7%) TDF-treated patients vs. 75/146 (51.4%) untreated patients. During follow-up, TE scores were available for 52/60 (86.7%) patients in the TDF treated group vs. 121/146 (82.9%) in the untreated group. Among these, 30/52 (57.7%) treated vs. 81/121 (66.9%) untreated patients had longitudinal measurements (at least two TE measurements). In the TDF group, TE score was 7.5 ± 5 (median ± IQR) kPa at baseline, and declined to 6.0 ± 1.5 kPa at four years (Fig. 3A, left panel), while in the untreated group the TE scores were 5.0 ± 2.0 kPa at baseline and at most subsequent time points during follow-up, however elevated TE scores were observed in particular patients (Fig. 3A, right panel). In the TDF group, elastography stage regressed over time, whilst a progression to F3 or F4 stages presented at 18, 30, 36, 42 months in the untreated group (Fig. 3B).

The crude mixed effects model revealed that changes in TE scores over time were significantly different for TDF-treated and untreated groups (*p* = 0.006, Table 4), with a decrease over time in the TDF-treated group (β_time + 1*β_interaction = − 0.032) but not in the untreated group (β_time + 0*β_interaction = 0.006). Similar longitudinal trends of TE scores were observed based on the model adjusted for demographics (*p* = 0.004, Table 4) and sensitivity analysis with further adjustment for baseline VL and ALT (*p* = 0.008, Table S4).

**Table 4:** Changes of TE scores (kPa) over time assessed by linear mixed effects models for patients treated with TDF vs. untreated

	Crude model		Adjusted for baseline age, gender, ethnicity	
	Coefficient β (95% CI)	***p***-value	Coefficient β (95% CI)	***p***-value
(Intercept)	5.088 (4.693, 5.483)	**< 0.001**	4.586 (3.385, 5.787)	**< 0.001**
Group	1.758 (0.977, 2.540)	**< 0.001**	1.729 (0.926, 2.533)	**< 0.001**
Time	0.006 (− 0.008, 0.020)	0.416	0.006 (− 0.008, 0.020)	0.421
Group x Time ^**a**^	−0.038 (− 0.065, − 0.011)	**0.006**	−0.040 (− 0.067, − 0.013)	**0.004**

^**a**^ x indicates the interaction between group and follow-up time. Group = {0,1}, where 0 indicates untreated, 1 indicates TDF. *CI* confidence interval; *TDF* Tenofovir disoproxil fumarate

There was no evidence of progressive liver fibrosis in the TDF group (Fig. S5A), whereas in the untreated group, 90.1% (73/81) of patients were stage F0-F1 at baseline, of whom 6/81 (7.4%) progressed (four of these to F2 and two to F3) (Fig. S5B). However, the difference in progression between the two groups did not reach statistical significance (0/30 vs. 6/81, *p* = 0.2). Among untreated individuals, the baseline TE reading (median ± IQR) was 6.0 ± 1.0 kPa in the subgroup with progressive liver fibrosis vs. 5.0 ± 2.0 kPa in the subgroup without (*p* = 0.09), and the former subgroup was older (median ± IQR, 45 (29-47) years vs. 35 (30-42) years, *p* = 0.5) (Table S5). These differences were not statistically significant, and due to small numbers we were underpowered to detect true associations with confidence (Table S5).

According to EASL or NICE guidelines [2, 7], two (2/6, 33.3%) of the untreated patients who experienced progression of fibrosis met treatment criteria at a later time. One met only EASL criteria at 9 months whilst not meeting NICE criteria - this patient was followed up to 12 months and did not receive treatment during follow-up. Another patient met both EASL criteria and NICE treatment criteria at 40 months (due to TE increasing to 12 kPa at this time) but with no further follow-up data after 40 months. However, the remaining four (4/6, 66.7%) still did not meet EASL or NICE criteria for treatment over the remaining follow-up period.

### No statistical difference in HBeAg and HBsAg loss rate between TDF treated and untreated groups

A small number of patients (*n* = 9) were HBeAg-positive at baseline (5 in the TDF group vs. 4 in the untreated group). HBeAg loss (seroconversion) occurred in 3/5 (60%) in the TDF group and 4/4 (100%) patients in the untreated group (one untreated patient also had HBsAg loss). HBsAg loss was recorded in 0/60 patients in the TDF group vs. 7/146 (5%) patients in the untreated group. Neither of these differences reached statistical significance (*p* = 0.4 for HBeAg loss and *p* = 0.2 for HBsAg loss).

### No significant difference in mild/moderate renal impairment over time between two groups and similar risks of CKD progression over time

**Fig. 4:**
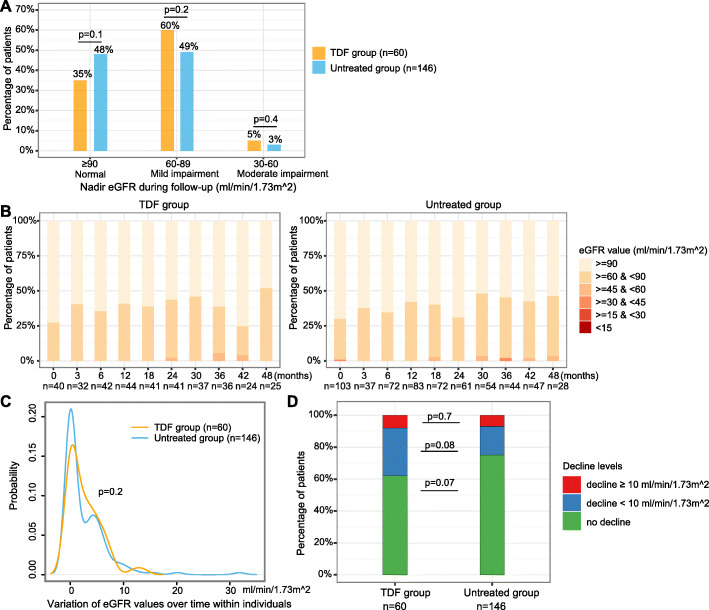
Longitudinal analysis of eGFR of chronic HBV patients with TDF treatment vs. without treatment. (**A**) Comparison of the proportion of patients with different categories of nadir eGFR during follow-up between groups; (**B**) Distribution of CKD stages at each time point; (**C**) Variation of eGFR values within individuals over time; (**D**) Stratifications of eGFR decline levels within individuals over time. *In panel A, nadir* eGFR *is defined as the lowest levels of eGFR of a patient during follow-up. In panel C, variation in the x-axis is calculated as the standard deviation of eGFR values of each individual during the follow-up period, y-axis is the probability of patients having a corresponding variation. eGFR, estimated Glomerular Filtration Rate; CKD, chronic kidney disease; TDF, Tenofovir disoproxil fumarate*

At baseline, 11/40 treated vs. 31/103 untreated patients had eGFR < 90.0 ml/min/1.73m^2^ (27.5% vs. 30.1%, *p* = 0.8), and 0/40 treated vs. 1/103 untreated patients had eGFR < 60 ml/min/1.73m^2^ (0 vs. 1%, *p* = 1) (Table 1). During follow-up, mild renal impairment (nadir eGFR 60–89 ml/min/1.73m^2^) was present in 60% (36/60) in TDF group vs. 49% (71/146) in the untreated group (*p* = 0.2), and moderate renal impairment (nadir eGFR 30–59 ml/min/1.73m^2^) in 5% (3/60) in TDF group vs. 3% (4/146) in untreated group (*p* = 0.4) (Fig. 4A, B**)**.

Using mixed effects regression analysis, the changes of eGFR over time were not significantly different between the TDF-treated and untreated groups based on both the crude model (*p* = 0.278) and the model adjusted for demographics (*p* = 0.268, Table 5). This was consistent in sensitivity analysis with further adjustment for baseline VL, ALT, and TE scores (*p* = 0.659, Table S6).

**Table 5:** Changes of eGFR (ml/min/1.73m^2^) over time assessed by linear mixed effects models for patients treated with TDF vs. untreated

	Crude model		Adjusted for baseline age, gender, ethnicity	
	Coefficient β (95% CI)	***p***-value	Coefficient β (95% CI)	***p***-value
(Intercept)	85.813 (84.676, 86.950)	**< 0.001**	93.974 (90.352, 97.595)	**< 0.001**
Group	0.991 (− 1.085, 3.068)	0.349	1.76 (− 0.286, 3.805)	0.092
Time	− 0.014 (− 0.050, 0.022)	0.449	−0.016 (− 0.051, 0.019)	0.379
Group x Time ^**a**^	−0.034 (− 0.094, 0.027)	0.278	−0.033 (− 0.092, 0.026)	0.268

^**a**^ x indicates the interaction between group and follow-up time. Group = {0,1}, where 0 indicates untreated, 1 indicates TDF. *CI* confidence interval; *eGFR* estimated glomerular filtration rate; *TDF* Tenofovir disoproxil fumarate

Variation in eGFR within individuals over time was not significantly different between the groups (*p* = 0.2) (Fig. 4C). We observed a decrease in eGFR of ≥ 10 ml/min/1.73m^2^ in 8% (5/60) of patients in TDF group and 7% (10/146) of patients in untreated group (*p* = 0.7, red sections in Fig. 4D), a decrease in eGFR of < 10 ml/min/1.73m^2^ in 30% (18/60) in TDF group vs. 18% (26/146) in untreated group (*p* = 0.08, blue sections in Fig. 4D). There was no significant difference in the proportion of patients with progression of CKD stages between the TDF group and untreated group (28.3% (17/60) vs. 17.8% (26/146), *p* = 0.13) (Fig. S6A, S6B).

### Risks of AKI events are not associated with TDF treatment

**Fig. 5:**
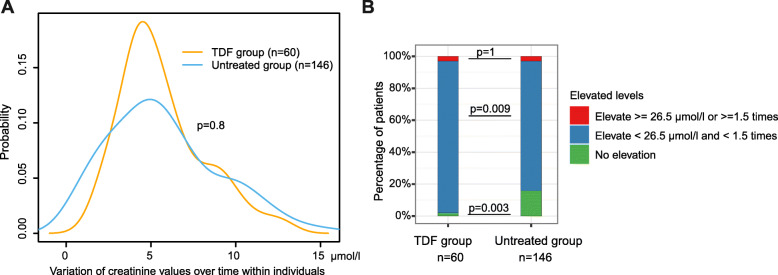
Changes of serum creatinine within individuals over time in chronic HBV patients with TDF treatment vs. without treatment. (**A**) Variation of serum creatinine levels within individuals over time; (**B**) Stratifications of serum creatinine elevation levels within individuals over time. *In panel A, variation in the x-axis is calculated as the standard deviation of creatinine values of an patients during the follow-up period, y-axis is the probability of patients having a corresponding variation. In panel B, red sections represent the percentage of patients had AKI events, which is defined as an elevation of serum creatinine of ≥ 26.5* μmol*/l or ≥ 1.5 times compared to previous time point. AKI, acute kidney injury. TDF, Tenofovir disoproxil fumarate*

There was no significant difference in variation of serum creatinine within individuals over time between the groups (*p* = 0.8) (Fig. 5A). AKI events arose in 2/60 (3.3%) of TDF-treated patients and 5/146 (3.4%) untreated patients (*p* = 1, Fig. 5B). We also observed a mild elevation of serum creatinine of < 26.5 μmol/l and < 1.5 times in 95% (57/60) in TDF group vs. 81% (118/146) in untreated group (*p* = 0.009, Fig. 5B). However, the median (IQR) creatinine in both groups was within the normal reference range, i.e., 81 (69-93) μmol/l in TDF group vs. 79 (66-89) μmol/l in untreated group (*p* = 0.2), with a small proportion who had creatinine elevated to a level > ULN (5/57 (8.8%) in TDF group vs. 5/118 (4.2%) in untreated group, *p* = 0.3); all of these ten patients had eGFR < 90 ml/min/1.73m^2^.

## Discussion

### Principal findings

In this longitudinal cohort study, using an electronic pipeline developed by NIHR HIC, we show that CHB patients treated with TDF therapy suppress HBV viraemia as expected, and this is associated with an improvement in liver stiffness when assessed up to ~ 5 years. Liver disease progressed in 7.4% of patients in the untreated group over the study period and in none of the TDF treated patients. Although this difference did not reach statistical significance, this observation demonstrates evolving liver disease among a subgroup of untreated patients, highlighting that broadening treatment criteria could be of benefit in preventing liver disease in a wider pool of the CHB population. This finding warrants exploration in larger cohorts followed up over longer time periods.

### Value added to the existing literature and clinical implications

There is currently minimal evidence assessing the development or regression of liver disease and changes in renal function in CHB patients receiving TDF therapy in comparison to an untreated group – especially in those without cirrhosis at baseline [12]. A significantly higher treatment rate in Asian patients (who had higher ALT levels and HBV VL at baseline compared to other ethnicities) is consistent with findings from previous studies that genotype C (common in Asia) is more likely to be HBeAg positive for longer, with higher HBV VL [45–47]. Therefore Asian patients are more likely to meet current treatment criteria, though we acknowledge that ethnicity was unreported for ~ 23% of patients in our cohort.

Our research has potential implications for clinical practice. Firstly, since TDF therapy is associated with disease regression in those without cirrhosis, treatment may be of benefit to a greater proportion of this population. Follow-up is often irregular, and is subject to clinical practice and patient adherence. Due to the irregularity of follow-up, fibrosis progression may occur months to years before detection. Wider treatment at the initial assessment might be advantageous compared to treatment initiation only after the progression of fibrosis has been observed. In this study, most of the untreated patients who experienced progression of liver fibrosis still did not meet treatment criteria at a later time according to EASL or NICE guidelines. Therefore, expanding guidelines to offer treatment to more individuals earlier in the course of infection might be of benefit in such patients for preventing liver disease progression.

Secondly, concerns about renal toxicity should not limit access to treatment; we found that untreated CHB patients also had a risk of CKD progression (consistent with a previous cohort study [28]), which was not significantly different from the risk in treated patients. Previous studies found that risk factors associated with renal function decline in CHB patients include old age, hypertension, diabetes, baseline impairment in eGFR, and use of diuretics [48–51]. Therefore, concerns about nephrotoxicity may be more pertinent in these subgroups, and risks should be assessed on a case-by-case basis.

A meta-analysis confirms that TDF and tenofovir alafenamide (TAF) are the most effective agents for virologic suppression for both HBeAg-positive and HBeAg-negative CHB patients [52], and promising short-term outcomes of TAF have been reported in several studies indicating reduced nephrotoxicity [53–55]. However, a recent study of cost-effectiveness of TAF for treatment of CHB in Canada reported that TAF is not cost-effective at its current cost with a price of more than four times that of TDF, without any advantage in efficacy [56]. Hence, TDF is currently still considered the treatment of choice. For special groups with baseline renal dysfunction or comorbidities, however, TAF may be a safer alternative to TDF [23, 24], but long-term efficacy and safety outcomes of TAF need to be confirmed [57, 58].

### Caveats and limitations

The power of our analysis is constrained by sample size, especially for identifying associations of uncommon outcomes (e.g. loss of HBsAg and progression of fibrosis in untreated patients). Although our population is ethnically diverse, the number of individuals in each group is small, and the data provides a snapshot of disease in one UK centre. We recognise that some missing data limits our ability to perform comparison analysis, e.g. missing HBeAg status by ethnic group. It will be useful to explore the HBeAg-positive rate by ethnic group when more data become available in the future. We reduced missingness at those time points of interest through data imputation using closest time points within a short period, and performed sensitivity analysis in a subset of patients who had no missing data for important covariates at baseline. It would be desirable to perform an assessment of the fractional urinary excretion of phosphate, albuminuria, and proteinuria to assess tubulopathy in future analyses.

We only considered adult patients and no data for special groups including pregnant women, or those with chronic comorbidities such as hypertension, diabetes, or co-infection with other blood-borne viruses. Therefore, we may have underestimated treatment impact or risk at a population level. Similar studies are needed in settings where other risk factors are more prevalent, e.g., populations where HIV is co-endemic. Previous investigation from South Africa reported that adults with HIV/HBV coinfection on antiretroviral therapy have less chronic liver disease than those with HBV monoinfection, suggesting an advantage conferred by treatment in the coinfected group [59]. We only compared patients on TDF treatment to untreated; due to infrequent use of ETV, we have not considered the small subgroup of our population treated with this agent. Based on the available data, we were not able to make a formal assessment of metabolic bone disease in patients on TDF therapy, nor could we consider other reported side-effects such as gastrointestinal disturbance. For expanding treatment eligibility, assessment of cost effectiveness would be also necessary in future studies, and the treatment duration or the necessity of life-long intake of TDF would need to be further evaluated.

## Conclusions

In summary, we show clear benefits of TDF therapy in an ethnically diverse CHB population, even among patients with only mild/moderate liver disease at baseline. These benefits may be relevant to a wider pool of the untreated CHB population without imposing clinically significant renal impairment in the time frames observed here. Studies of larger populations, and over longer periods of follow-up, are urgently needed to provide the evidence to underpin expanded treatment guidelines.

## Supplementary Information

**Additional file 1.**

## Data Availability

The datasets supporting the findings of this article are available in this manuscript and the supplementary material.

## Abbreviations

AKI: Acute kidney injury
ALT: Alanine transaminase
anti-HBe: Antibody to HBeAg
CHB: Chronic HBV
cccDNA: Covalently closed circular DNA
CKD: Chronic kidney disease
ETV: Entecavir
eGFR: Estimated glomerular filtration rate
EASL: European Association for the Study of the Liver
HBV: Hepatitis B virus
HBeAg: HBV e antigen
HBsAg: HBV surface antigen
HCC: Hepatocellular carcinoma
HCV: Hepatitis C virus
HDV: Hepatitis delta virus
K-M: Kaplan-Meier
NIHR HIC: National Institute for Health Research Health Informatics Collaborative
OUH: Oxford University Hospitals
NHS: National Health Service
SD: Standard deviation
SQL: Structured query language
TDF: Tenofovir disoproxil fumarate
TE: Transient elastography
ULN: Upper limit of normal
VL: Viral load
WHO: World Health Organisation.
